# Correlation Between Prediabetes Conditions and Microalbuminuria

**DOI:** 10.5812/numonthly.7646

**Published:** 2013-03-30

**Authors:** Adele Bahar, Atieh Makhlough, Atefe Yousefi, Zahra Kashi, Saeid Abediankenari

**Affiliations:** 1 Diabetes Research Center, Mazandaran University of Medical Sciences, Sari, IR Iran

**Keywords:** Glucose Tolerance, Diabetic Nephropathies, Diabetes Mellitus, Type 2

## Abstract

**Background:**

Impaired fasting glucose (IFG), and impaired glucose tolerance (IGT) are two prediabetes conditions which have some correlation with macrovascular disorders. The risk of microvascular complications in these groups is not clear.

**Objectives:**

The prevalence of albuminuria in subjects with IFG and IGT was evaluated in the present study.

**Patients and Methods:**

In this study three groups of subjects were entered (45 subjects in each group): IFG, IGT, and normal glucose tolerance as control. The urine albumin-creatinine ratio was studied in morning spot urine samples to detect microalbuminuria. The subjects were followed up for two years, and blood sugar and urine albumin and glycosylated hemoglobin (HbA1C) were measured every 6 months.

**Results:**

The prevalence rate of microalbuminuria was 15.5% in the prediabetic groups, while no one had microalbuminuria in the control group (P = 0.005). The prevalence of microalbuminuria in patients with IFG or IGT was not significantly different (17.8% vs. 13.3%) (P = 0.4). Fourteen subjects (4 in IFG group and 10 in IGT group) developed diabetes mellitus within a 2-year follow-up period (P = 0.1). Thirty six percent of subjects with albuminuria, and twelve percent of subjects without albuminuria progressed to diabetes mellitus during a 2-year follow-up (P = 0.02, odd ratio = 4.1; CI95%, 1.13-15.1).

**Conclusions:**

The risk of microalbuminuria in prediabetic subjects is high, and probably prediabetic subjects are at higher risk of progression to diabetes mellitus. We suggest periodically evaluation of albuminuria in prediabetic patients after the diagnosis.

## 1. Background

Impaired fasting glucose (IFG), and impaired glucose tolerance (IGT) are two forms of abnormal glucose metabolism which are located between the normal glucose tolerance (NGT), and type 2 diabetes. These two disorders have significant pathophysiological effects on insulin sensitivity, and secretion as well as cardiovascular diseases (1, 2). Epidemiological studies have considered microalbuminuria (MAU) as a risk factor for atherosclerosis, coronary artery disease, and other vascular disorders in patients with type 2 diabetes, and IGT (3-5). Microalbuminuria refers to a slight increase in secretion of albumin in urine, and is a sign of progression towards nephropathy in patients with diabetes (6). It is a clue which helps us predict the occurrence of cardiovascular disorders in both patients with or without diabetes (7, 8). The risk of microalbuminuria is correlated with plasma glucose level, and the duration of hyperglycemia in patients with diabetes (9, 10). Glycemic control in these patients can prevent the development, and progression of microalbuminuria, but this issue has not been well-documented about IGT and IFG-related disorders yet (11). Some studies conducted in this regard have shown that IGT is a more important risk factor than IFG for developing microalbuminuria (5). At present, no special treatment and diagnostic measures is advised in patients with IGT and IFG.

## 2. Objectives

Considering that early diagnosis and control of microalbuminuria can slow its progression towards macroalbuminuria and renal failure, and also prevent cardiovascular complications, the present study was designed to investigate the probable correlation between microalbuminuria and IFG and IGT.

## 3. Patients and Methods

This cohort study was performed from 2009 to 2011 in university hospital of Mazandaran university of medical sciences, Sari, Iran. Exclusion criteria were as follows: overt diabetes mellitus (12), hypertension, heart failure, renal failure, previous history of proteinuria, recent urinary tract infection (UTI), and treatment with corticosteroids or spironolactone, ARBs (angiotensin receptor blockers), and ACEIs (angiotensin converting enzyme inhibitors). The study population was sequentially selected based on the results of glucose tolerance test (GTT, 75 g), and the level of FBS. FBS ≥ 100mg/dL and less than 126 was considered as IFG. IGT was defined between 140-199 mg/dL of serum glucose after two-hour oral 75 g glucose consumption. The study was approved by the local ethics committee, and informed consent was obtained from all participants.

The quantitative urine albumin-creatinine (Cr) ratio in morning spot urine samples were used for standard microalbuminuria determination. For these measurements, the automated clinical chemistry analyzer by immunotorbidometry assay with prstige24i device (auto analyzer, Japan), Pars Azmon Kit (Iran) were used. microalbuminuria was defined as 30-300 mg/g Cr (13) in two random measurements with a month interval.

The subjects with different results in these two random tests (one positive and one negative) were asked for testing the third sample. All of our cases were followed up for two years, we did not have any drop out in our patients follow, and their blood sugar and urine albumin levels were measured every 6 months. For each participant, HbA1C was requested at least two times in a two-year follow up. We used chromatography method with Bio-system Kit (Italy, CV = 5%) for this test.

The obtained data was analyzed by SPSS software. ANOVA, X2 and fisher exact tests were used for comparison of data between the groups. Correlation between blood glucose and urine albumin was evaluated by the Pearson correlation coefficient. P value less than 0.05 was considered as statistically significant.

## 4. Results

The mean (SD) age of the subjects was 52.5 (± 11.2), 51.4 (± 12.2) and 40.2 (± 12.0) years in the IFG, IGT and control groups respectively (P < 0.001). Of forty five subjects with normal glucose tolerance, 12 (26.7%) were male, and 33 (73.3%) were female. Thirty four (76.6%) of subjects with IFG, and 38 (84.4%) with IGT were female (P = 0.4). Basic fasting blood sugar and blood glucose 2 hours after 75 gr oral glucose in three groups (IFG, IGT and control groups) were shown in Table 1.

**Table 1.** The Basic Data in Study Population

	IFG ^a^	IGT ^a^	NGT ^a^
FBS (mg/dL) ^b^	95.5 ± 108.11	8.36 ± 110.36	75.31 ± 5.82
BS2h G (mg/dL) ^b , c^	19.19 ± 105.42	17.4 ± 160.36	68.73 ± 5.70
BMI (kg/m^2^) ^c^	29.37 ± 4.72	30.15 ± 4.41	26.15 ± 3.72

^a^Abbreviations: IFG, Impaired fasting glucose; IGT, impaired glucose tolerance; NGT, normal glucose tolerance

^b^P < 0.001 between three groups

^c^P < 0.001 between IFG and IGT group

**Figure 1.**
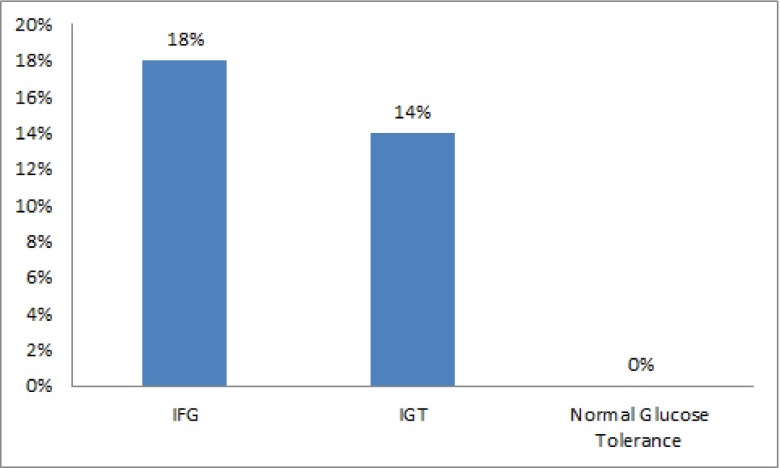
Microalbuminuria Prevalence in Prediabetes Conditions, and Normal Population

Microalbuminuria was not seen in the control group. The prevalence rate of microalbuminuria was 15.5% in prediabetic subjects (P = 0.005). The prevalence of microalbuminuria in patients with IFG or IGT was not significantly different (P = 0.4) (Figure 1).

**Figure 2.**
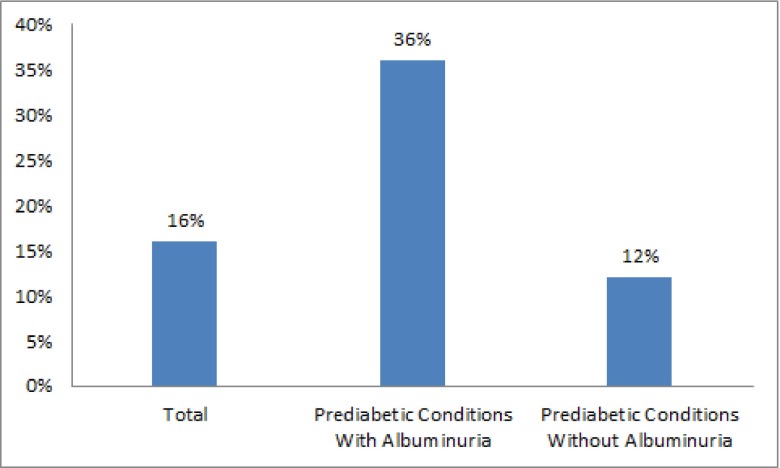
Progression Toward Diabetes Mellitus in Subjects With Prediabetes Conditions

Urine albumin secretion in both samples measurement was significantly correlated with FBS (r = 0.32, P < 0.0001; r = 0.3, P < 0.001; r = 0.32, P < 0.0001), and BS2hpp (r = 0.3, P = 0.002; r = 0.3, P = 0.001). Fourteen subjects (4 in IFG group and 10 in IGT group) developed diabetes mellitus within a 2-year follow-up period (P = 0.1). [according to the ADA 2011 definition of diabetes mellitus (14)]. Prediabetic subjects with albuminuria were four times at risk of progression to diabetes mellitus than subjects without proteinuria during a 2-year follow-up (P = 0.02, Odd ratio = 4.1; CI95%, 1.13-15.1) (Figure 2). The distribution of diabetes mellitus in albuminuric subjects with IFG and IGT were 7.1% and 28.6 % respectively (P = 0.03).

## 5. Discussion

This study investigated the presence of microalbuminuria in patients with IFG and IGT, and compared them to the control group (NGT). The prevalence of microalbuminuria in prediabetic subjects was significantly higher than normal glucose tolerance cases, but the prevalence of microalbuminuria was not significantly different between the two groups with IFG and IGT. In Monica study on Italian subjects, the prevalence of microalbuminuria were 6.9%, 5.6%, and 4.3% in IFG, IGT and NGT groups, respectively (15). The prevalence of microalbuminuria was 8.3% in IFG, 9.9% in IGT, and 4.3% in NGT groups in Robyn study in Australia (16). The difference in prevalence reported by different studies can be attributed to the differences in population indexes such as race, laboratory techniques for urine albumin measurement, and the differences in the definition of microalbuminuria, IFG, and diabetes mellitus.

The current study revealed that the urine albumin secretion is significantly higher in the IFG and IGT groups than in the NGT group. Although the prevalence and risk of microalbuminuria was higher in IFG group than in IGT group, this difference was not statistically significant in our study. Meigs in Framingham Offspring Study reported a strong correlation between IFG and microalbuminuria (11). In Wang study the prevalence of microalbuminuria in IGT group was higher than IFG group, and IGT was introduced as the most important risk factor for microalbuminuria (5). The mechanism of albuminuria in relation with glycemia involves the glycation of basal membrane proteins, losing the selective power of membrane, glomerular hyperperfusion, and its hyperinfiltration (17). Also, it has been revealed that the relation between hyperglycemia and microalbuminuria is independent from the blood pressure (5). In our study, the cases with hypertension were excluded from the study. Fourteen subjects (10.4%) in our study developed diabetes within a 2-year follow-up were 4 in the IFG, and 10 in the IGT groups respectively. This suggests that the risk of diabetes was greater for the IGT group, whereas our study revealed more correlation between IFG and risk of microalbuminuria. The limitation of our study was that we did not repeat the glucose tolerance test during the two year follow up for diabetes mellitus diagnosis, although we measured HbA1C for this mention (14). In our study the body mass index has not been matched between the three groups; despite the fact, according to a large sample study with 20,828 cases by Thoenes, BMI had not any association with albuminuria (18).

Our study showed that the prevalence of microalbuminuria is high in prediabetes conditions such as IFG and IGT, and it correlates with the progression towards diabetes mellitus in this population. We recommend periodic evaluation of urine albumin in addition to HbA1C in patients with prediabetes conditions.

## References

[1] Hanefeld M, Koehler C, Fuecker K, Henkel E, Schaper F, Temelkova-Kurktschiev T (2003). Insulin secretion and insulin sensitivity pattern is different in isolated impaired glucose tolerance and impaired fasting glucose: the risk factor in Impaired Glucose Tolerance for Atherosclerosis and Diabetes study.. Diabetes Care..

[2] Weyer C, Tataranni PA, Bogardus C, Pratley RE (2001). Insulin resistance and insulin secretory dysfunction are independent predictors of worsening of glucose tolerance during each stage of type 2 diabetes development.. Diabetes Care..

[3] Dell'Omo G, Penno G, Giorgi D, Di Bello V, Mariani M, Pedrinelli R (2002). Association between high-normal albuminuria and risk factors for cardiovascular and renal disease in essential hypertensive men.. Am J Kidney Dis..

[4] Garg JP, Bakris GL (2002). Microalbuminuria: marker of vascular dysfunction, risk factor for cardiovascular disease.. Vasc Med..

[5] Wang XL, Lu JM, Pan CY, Tian H, Li CL (2005). A comparison of urinary albumin excretion rate and microalbuminuria in various glucose tolerance subjects.. Diabet Med..

[6] Fioretto P, Caramori ML, Mauer M (2008). The kidney in diabetes: dynamic pathways of injury and repair. The Camillo Golgi Lecture 2007.. Diabetologia..

[7] Mogensen CE (1984). Microalbuminuria predicts clinical proteinuria and early mortality in maturity-onset diabetes.. N Engl J Med..

[8] Yudkin JS, Forrest RD, Jackson CA (1988). Microalbuminuria as predictor of vascular disease in non-diabetic subjects. Islington Diabetes Survey.. Lancet..

[9] (1996). The absence of a glycemic threshold for the development of long-term complications: the perspective of the Diabetes Control and Complications Trial.. Diabetes..

[10] Nelson RG, Kunzelman CL, Pettitt DJ, Saad MF, Bennett PH, Knowler WC (1989). Albuminuria in type 2 (non-insulin-dependent) diabetes mellitus and impaired glucose tolerance in Pima Indians.. Diabetologia..

[11] Meigs JB, D'Agostino RB, Sr, Nathan DM, Rifai N, Wilson PW (2002). Longitudinal association of glycemia and microalbuminuria: the Framingham Offspring Study.. Diabetes Care..

[12] (2007). Diagnosis and classification of diabetes mellitus.. Diabetes Care..

[13] (2007). KDOQI Clinical Practice Guidelines and Clinical Practice Recommendations for Diabetes and Chronic Kidney Disease.. Am J Kidney Dis..

[14] (2011). Diagnosis and classification of diabetes mellitus.. Diabetes Care..

[15] Franciosi M, Pellegrini F, Sacco M, De Berardis G, Rossi MCE, Strippoli GFM (2007). on behalf of the IGLOO (Impaired Glucose tolerance, and Long-term Outcomes Observational Study) Study Group: Identifying patients at risk for microalbuminuria via interaction of the components of the metabolic syndrome: A cross-sectional analytic study.. Clin J Am Soc Nephrol..

[16] Tapp RJ, Shaw JE, Zimmet PZ, Balkau B, Chadban SJ, Tonkin AM (2004). Albuminuria is evident in the early stages of diabetes onset: results from the Australian Diabetes, Obesity, and Lifestyle Study (AusDiab).. Am J Kidney Dis..

[17] Tarsio JF, Reger LA, Furcht LT (1988). Molecular mechanisms in basement membrane complications of diabetes. Alterations in heparin, laminin, and type IV collagen association.. Diabetes..

[18] Thoenes M, Reil JC, Khan BV, Bramlage P, Volpe M, Kirch W (2009). Abdominal obesity is associated with microalbuminuria and an elevated cardiovascular risk profile in patients with hypertension.. Vasc Health Risk Manag..

